# The Role of Mobility and Sanitary Measures on the Delay of Community Transmission of COVID-19 in Costa Rica

**DOI:** 10.3390/epidemiologia2030022

**Published:** 2021-07-21

**Authors:** Luis A. Barboza, Paola Vásquez, Gustavo Mery, Fabio Sanchez, Yury E. García, Juan G. Calvo, Tania Rivas, María Dolores Pérez, Daniel Salas

**Affiliations:** 1 Centro de Investigación en Matemática Pura y Aplicada-Escuela de Matemática, Universidad de Costa Rica, San Pedro Montes de Oca 11501, Costa Rica; paola.vasquez@ucr.ac.cr (P.V.); fabio.sanchez@ucr.ac.cr (F.S.); ygarciapuerta@ucdavis.edu (Y.E.G.); juan.calvo@ucr.ac.cr (J.G.C.); 2 Pan America Health Organization, World Health Organization, San José 10102, Costa Rica; merygus@paho.org (G.M.); perezmd@paho.org (M.D.P.); 3 Department of Public Health Sciences, University of California, Davis, CA 95616, USA; 4 Ministry of Health, San José 10102, Costa Rica; tania.rivas@misalud.go.cr (T.R.); daniel.salas@misalud.go.cr (D.S.)

**Keywords:** public health, SARS-CoV-2, human mobility, change-point, bayesian estimation

## Abstract

The aim of this paper is to infer the effects that change on human mobility had on the transmission dynamics during the first four months of the SARS-CoV-2 pandemic in Costa Rica, which could have played a role in delaying community transmission in the country. First, by using parametric and non-parametric change-point detection techniques, we were able to identify two different periods when the trend of daily new cases significantly changed. Second, we explored the association of these changes with data on population mobility. This also allowed us to estimate the lag between changes in human mobility and rates of daily new cases. The information was then used to establish an association between changes in population mobility and the sanitary measures adopted during the study period. Results showed that during the initial two months of the pandemic in Costa Rica, the implementation of sanitary measures and their impact on reducing human mobility translated to a mean reduction of 54% in the number of daily cases from the projected number, delaying community transmission.

## 1. Introduction

The pandemic caused by the severe acute respiratory syndrome coronavirus 2 (SARS-CoV-2) has produced an unprecedented global sanitary crisis that has deeply disrupted societies and decimated economies.

After attempts to contain the virus failed and COVID-19 spread to practically every country in the world, nations implemented an arsenal of public health measures in an effort to avoid or at least delay community transmission. Such measures included a diverse range of lockdown-type interventions and different forms of business restrictions, which basically aim at limiting population mobility and therefore reducing physical contact among individuals outside their family nucleus [1,2].

As the pandemic unfolded, these measures had been lifted, reimposed, and modified multiple times in an effort to avoid the collapse of health services and to mitigate the number of deaths, at the same time allowing the return of different degrees of personal freedom and giving a break to struggling economies. In this ongoing effort to balance these two often opposing sides of the equation, the effectiveness of specific sanitary measures in controlling the spread of COVID-19 has been largely disputed [3,4,5]. The concurrence of numerous simultaneous sanitary measures, added to countless societal variables, makes determining the effect of every individual measure a nearly impossible task.

One way to study whether sanitary measures or sets of measures had a significant impact on disease transmission is through establishing their association with changes in population mobility and determining the impact of these changes in the speed of disease transmission.

Studies on the issue of human mobility were first focused on restrictive measures to avoid the geographic spread of SARS-CoV-2 in China [6,7] and on demonstrating associations in theoretical models [8,9].

Badr et al. [10] used United States mobile phone data to study this issue outside China. They were able to correlate mobility patterns with COVID-19 case growth rates. More recently, Cot et al. [11] showed strong decreases in infection rates using Google and Apple mobility data. The study focused on the effect of social distancing at the beginning of the pandemic in Europe and the United States. Cazelles et al. [12] reached similar conclusions in the period between the first and second waves in Europe using the effective reproduction number.

Costa Rica represents a distinctive setting to test these assumptions. In this particular Central American country, an initial effective control of the pandemic allowed avoiding community transmission for the first four months—between March and July 2020—when the virus was rapidly spreading in neighboring countries and almost across the whole Americas [13]. Moreover, Costa Rica never imposed a total lockdown but rather imposed restrictions to vehicular circulation combined with several restrictions to commercial activity, public gatherings and school closures [14]. This situation allowed us to test our hypotheses before community transmission was established and the detection of new cases became more challenging.

## 2. Materials and Methods

### 2.1. Data. The Analysis Uses Three Different Sources of Information

- Epidemiological data: The number of daily confirmed COVID-19 cases in Costa Rica from 6 March 2020 to 2 July 2020 was obtained from the Ministry of Health [14]. The data covers 4023 laboratory-confirmed cases from 82 municipalities across the country. On March 6, Costa Rica became the 89th country to confirm a COVID-19 patient within the national territory, a 49-year-old woman visiting from New York [14]. During the initial months of the pandemic, the growth of confirmed cases was relatively stable in Costa Rica, and the majority of patients had a known chain of transmission. Two months into the pandemic, the country was even highlighted as having the lowest COVID-19 case fatality rate in the region [15]. However, by the second week of June 2020, the daily new cases began a progressive growth. On 2 July 2020, with 4024 total confirmed cases, a 14-day case notification rate of 40.3 cases per 100,000 population and 17 deaths, the Ministry of Health declared community transmission in the Greater Metropolitan Area after being unable to establish the epidemiological nexus to 65% of all new positive cases detected over the last five days [14]. This region comprises about 50% of the national population.
- Google’s Community Mobility Reports [16]: This data contains relative changes of mobility according to Google’s applications with respect to the data observed on a certain baseline. The baseline day is the median value using a five-week period in January and February 2020. The relative changes are computed using the following categories: Retail and Recreation, Parks, Transit stations, Residential, Grocery and Pharmacy, and Workplaces.
- Sanitary measures: Since the first reported case of COVID-19 in Costa Rica, an inter-institutional and comprehensive approach has guided the country’s response to mitigate the impact of the COVID-19 pandemic. One of the first measures aimed to promote and facilitate physical distancing took place on 10 March 2020, with the cancellation of all massive events and the instruction of working from home [14]. In the days to come, the country restricted the capacity of public meeting spaces to 50%, closed schools and universities, all air and land borders, bars, beaches, churches, gyms, theaters, and cinemas [14]. By 23 March 2020, with a total of 158 confirmed cases spread across 30 municipalities (from a total of 82), public health authorities imposed the first restriction to vehicle circulation, beginning with a nighttime restriction to circulate from 10 p.m. to 5 a.m. [14]. The opening, closure, and allowed capacity for both commercial activities and social gathering places, as well as the vehicle restrictions, have been adjusted throughout the pandemic.

### 2.2. Methods

The methodological purpose of the study is three-fold. First, we determined significant changes in the speed of disease transmission before the community transmission started in Costa Rica. Second, we attempted to determine the validity of the association between population mobility within the national territory and the speed of disease transmission using a Bayesian approach in order to measure the impact of the detected changes. Third, we identified which measures were adopted when those changes in mobility occurred in order to explore the effectiveness of such sanitary measures in the control of the disease using MANOVA.

The estimation of time points where the series of newly infected cases changed in terms of trend, variability, or more distributional properties, can give an insight of the approximate days where a certain intervention measure, or a combination of several measures, impact the underlying behavior of the transmission dynamics. Those time points are commonly referred to in the statistical literature as change-points. In this effort, the detection of single and multiple change-points in the time series of new cases has been a way to infer the impact of interventions in different countries. For example, Dehning et al. combined Bayesian inference with compartmental models to identify plausible change-points on the COVID-19 spreading rate in Germany [17], and Jiang et al. used a novel combination of algorithms to detect multiple change-points in the series of confirmed cases and deaths of COVID-19 over more than 30 countries [18]. Recently, Coughlin et al. showed an interesting application of change-point detection in COVID-19 using the information of new cases in 20 individual countries (excluding Costa Rica) and the European Union in an aggregated way [19]. They were able to identify change-points in trend and variability using a Bayesian Change Point model together with a B-spline procedure. In order to estimate where those changes probably occurred in the series of newly infected cases, we use non-parametric and parametric change-point detection techniques. In the case of non-parametric, we used the Change Point model (CPM) for sequential multiple change detection introduced in [20,21], implemented in [22,23,24] and broadly used in different environmental applications, for example [25,26,27,28]. We used several hypothesis tests to apply the non-parametric procedure on the Change Point model detection: Mood [29], Lepage [30], and the well-known Kolmogorov–Smirnov, Mann–Whitney, and Cramer–von-Mises tests. In the case of a parametric sequential detection of phase changes through outlier identification according to [31] (Chen-Liu). The change-points detected are likely due to the impact of mobility restrictions or allowances on the days before the effect was quantified. A cross-correlation analysis among the rate of change of new cases and Google’s Mobility Trends allowed us to determine which lags are more significant under a Pearson hypothesis test.

To determine the association between changes detected in disease transmission and population mobility, we used a Bayesian Structural Time (BST) series model fitted with a Markov chain Monte Carlo (MCMC) sampling algorithm. The model was then combined with data from Google’s Community Mobility Reports to infer the cumulative difference between the observed and expected number of the projected cases after the change- points were detected. In this way, we attempt to combine the above information to infer the causal impact of the mobility preceding each change-point. Here we use a certain type of BST model called the Bayesian structural time-series model [32] with a set of K lagged covariates with significant association with respect to the dependent variable. The covariate lags can be determined by means of cross-correlation analysis. Two advantages of this model are that: (1) it is able to keep the temporal association in the modeling process without using the restrictive assumption of independence in observations and (2) the Bayesian approach combined with a state-space structure assures a natural way to propagate the uncertainty along the model hierarchy. In our case, the BST model can be written as:

(1) $$\begin{array}{c} y_{t}=\mu_{t}+x_{t}^{T}\beta +\sigma_{y}\varepsilon_{t}\\ \mu_{t}=\mu_{t-1}+\sigma_{\mu }\eta_{t} \end{array}$$

where *y_t_* are the new infections due to COVID-19 at day *t*, $x_{t}^{T}$ = $(M_{t-l1}^{\left(1\right)},$ $M_{t-l2}^{\left(2\right)}$, $M_{t-l_{K}}^{\left(K\right)})$ are lagged mobility covariates with their respective lags $l_{k}$(*k* = 1,…, *K*), *β* = ($\beta_{1},\ldots ,\beta_{K}{)}^{T}$, *σ_y_* and *σ_µ_* are parameters, *µ_t_* is a latent variable indicating the trend behavior of *y_t_*, and $\varepsilon_{t}$ and *η_t_* are normally-distributed white noises.

We use the MCMC algorithm to fit the model according to Equation (1), using the available data before each change-point occurred and assuming for the subsequent change-point that the data starts immediately after the previous change-point happened. The parameter set *θ* = (*β*, *σ_y_*, *σ_µ_*) is assumed to follow a spike-and-slab prior according to Equation 2.8 in [32]. The MCMC is performed with the R package CausalImpact [32], where posterior samples of the observed series and model parameters *θ* are computed. For each model, the time period can be divided into two different sets: a pre-intervention period containing the time before the change-point occurs and where the MCMC process is performed and a post-intervention period, where the MCMC process gives estimates of the observed series that we then compare with real values in order to approximate the causal effect of interventions.

In general, BST models provide methodological alternatives to include different sources of information together with a hierarchical structure of latent variables and error terms [33]. The BST approach during the estimation process provides a natural way to combine prior and sample information in the specification of the posterior distributions of both latent variables and parameters. In this sense, the MCMC algorithm helps to obtain those posterior distributions with many computational advantages. Bayesian and frequentist structural models have been widely used in epidemiological and environmental applications [34,35,36,37].

To explore associations between the variations detected in population mobility and the sanitary measures adopted by the health authorities, we identify all sets of measures imposed from March through June 2020, just before the appearance of the COVID-19 community transmission in Costa Rica. For simplicity, we assume that each set of measures is represented by an indicator at a unique date, chosen as the midpoint of the period where the set of measures is valid. We then apply a MANOVA model among the four mobility series (Retail and Recreation, Parks, Transit stations and Residential) and two temporal components: (1) a weekly seasonal effect and (2) a conditional effect of the set of measures given that the time of the previous set is taken as the start of the fitting set of observations. Finally, we apply a Pillai test to infer the effect of each individual set of measures given its preceding set to quantify its conditional impact on the mobility series. In this way, we were able to associate the set of measures with the mobility information to deduce their qualitative impact on the number of cases of COVID-19.

## 3. Results

**Figure 1:**
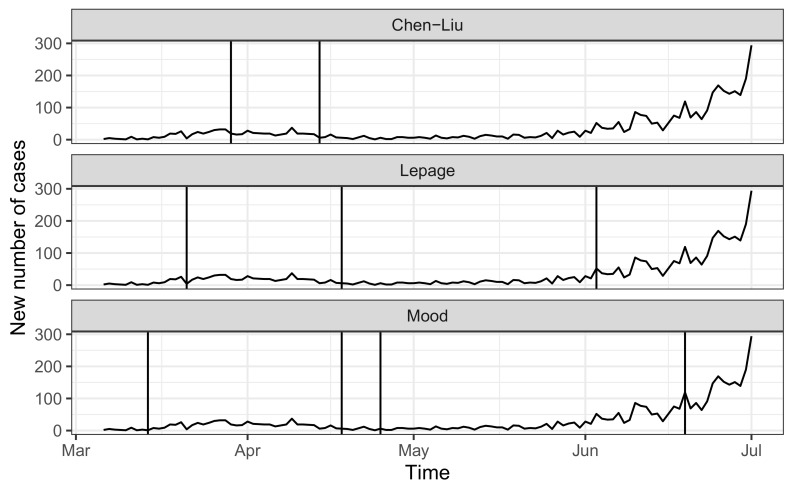
Estimated change-points (vertical lines) of new COVID-19 infected cases, comparing between the three methods.

Using the change-point for sequential multiple change detection, we detected significant points with the non-parametric Mood [29] and Lepage [30] tests using the series of log-differences of newly infected cases as a way to quantify the rate of change of the overall series. Figure 1 shows the series of new infected COVID-19 cases with the estimated change-points with the available methodologies.

Note that all the procedures were able to identify at least three different periods where the distributional properties of the newly infected cases significantly changed. Since the methods detect different elements, the periods identified are not the same, but when combined, they show a larger consensus that the series of newly infected cases experiences change-points in dates between 14 April 2020 and 18 April 2020 and between 3 June 2020 and 19 June 2020. For ease of analysis, we chose 18 April 2020 and 19 June 2020 as the estimated dates where the change-points occurred.

A cross-correlation analysis among the rate of change of new cases during mid-April and mid-June 2020 and Google’s Mobility Trends allowed us to determine that seven-to-eight-day lags are more significant under a Pearson hypothesis test, as shown in Table 1.

We then fit two BST models. The first one assumes that 18 April 2020 is a change point, and the second one does the same with 19 June 2020, using the MCMC procedure with 1000 samples. We used the set of Google’s mobility series along with their lags as covariates.

**Figure 2:**
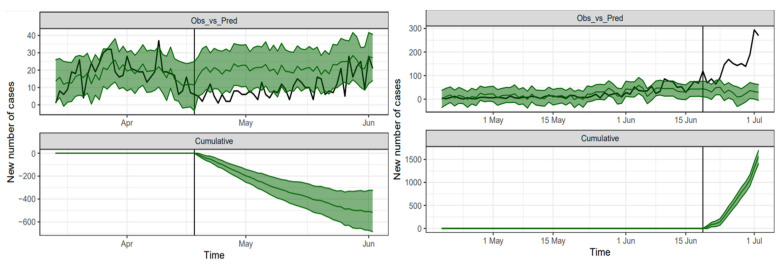
Upper panels: Bayesian estimation (green line and green shaded 95% credible region) of the observed number of COVID-19 cases (black line) during the study periods: 14 March–2 June 2020 (**left**) and 18 April–2 July 2020 (**right**). Lower panels: the estimated cumulative effect of the measures taken before 18 April 2020 (**left**) and 19 June 2020 (**right**).

The upper panels of Figure 2 contain the Bayesian estimation of the observed process for both periods together with their respective 95% predictive regions. The lower panels contain the posterior estimation of the cumulative effect of the intervention along the post-intervention period.

The upper-left panel in Figure 2 shows the Bayesian estimation of the causal effect using 14 March 2020 through 18 April 2020 data as the pre-intervention period and 19 April 2020 through 2 June 2020 as the post-intervention period. We remark that between 342 and 697 cases represent a 95% confidence interval of the cumulative effect on 2 June 2020 of the set of interventions before 18 April 2020, with a mean value of 526 cases. This mean value approximately represents 47% of the observed cumulative cases on 2 June 2020. The daily difference between the expected (under the pre-intervention conditions) and the observed number of cases is between 6 and 15 cases with the same confidence level, with a mean difference of 11 cases. In relative terms, there is a mean reduction of 54% in the number of daily cases with a confidence interval of (35%, 71%) approximately.

The upper-right panel in Figure 2 shows the fitting with 19 April 2020 through 19 June 2020 as pre-intervention period and 20 June 2020 through 2 July 2020 as post-intervention period. In this case, the effect is the opposite. There is a mean cumulative difference between observed and estimated cases of 1965 cases on 2 July 2020. This represents 48.8% of the observed cumulative cases on the same date. The daily difference among the observed and the expected number of cases (under the pre-intervention conditions) is between 109 and 131 cases with the same confidence level, with a mean difference of 120 cases. In relative terms, there is a mean increase of 387% in the number of daily cases with a confidence interval of (351%, 423%) approximately.

**Figure 3:**
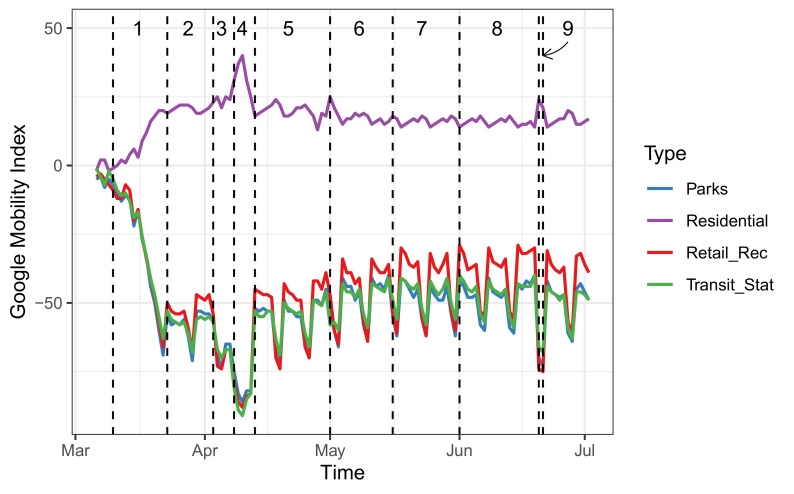
Google mobility indices (solid lines) along with the sets of sanitary and mobility measures (dashed lines). The sets of measures are defined in Table 2. Abscissa: Time (Days), Ordinate: Daily Google Mobility Index.

Table 2 contains the sets of measures from 10 March 2020 to 21 June 2020, just before the community transmission started in Costa Rica. The conditional effect of each set of measures is shown in the fourth column, measured by the p-value of the Pillai test. Note that all the sets cause a significant effect on the group of mobility covariates when we consider the weekly effect of mobility patterns. However, the effect of mobility with respect to sanitary measures varies across time periods. The first two sets are expected to show a larger effect because of the abrupt change in mobility behavior during March 2020 (see Figure 3). In these initial periods, although sanitary measures greatly impacted human mobility, they are not expected to correlate with significant changes in disease transmission because infected cases were still too low. Afterward, the conditional effect of additional measures is smaller, but set 4 shows a larger impact due to the general mobility restriction during the period between April 8 and 12 (Holy Week in Costa Rica). Starting from set 5, the subsequent mobility openings had a significant impact on Google’s indices, and the effect tends to decline by the end of June 2020. That is, the effect of lifting sanitary measures significantly increased the mobility in the period associated with an increase in newly reported cases.

## 4. Discussion

As the transmission of the SARS-CoV-2 virus has progressed around the world, a primary focus of decision-makers has been to implement comprehensive public health measures adapted to each country’s unique capacities and context [38]. Our findings show several conclusions that may prove useful to inform on the effectiveness of some of such measures to slow down the transmission of the disease or the role that they may have had in delaying community transmission.

First, through the use of change-point detection algorithms, we were able to identify two periods where the tendency of cases significantly varied. Then BTS models found a direct association between human mobility and this reduction in the transmission rate of the disease. After the first detected change-point on 18 April 2020, we were able to estimate a mean reduction of 54% in the number of daily cases from the projected number. We identified an association between some sanitary measures and this significant variation in human mobility patterns. Based on the estimated lag period of seven days and the high correlation estimated by the Pillai test, this reduction in the number of cases can be traced back to the set of sanitary measures taken by the health authorities during Holy Week, from 3 April 2020 to 12 April 2020 [14], in which the circulation of vehicles and public transportation was suspended. This finding proves wrong arguments against restrictions in vehicular circulation as not effective at reducing the transmission of SARS-CoV-2 in Costa Rica.

During March and April 2020, the epidemiological context of the country was led by well-established clusters of cases and a slow increase in the number of daily cases. However, the country was also in a race to increase hospital capacity, mainly for intensive care units. This health crisis drove the Ministry of Health to implement restrictive measures in order to flatten the curve of new cases, to avoid the saturation of health care services, and to give time to health officials to increase the human resources, hospital beds, ventilators, and personal protective equipment necessary to care for the patients.

It is also noteworthy that the effect of these restrictions was significant even compared to the previous period, when human mobility was already reduced due to major sanitary measures, such as the cancellation of massive events, school closures, and restrictions on all non-essential commercial activities. Indeed, in 2021, Costa Rica has seen the largest surge in cases and hospital admissions so far; this led to health authorities imposing restrictions in commercial activity and school closures. In light of the research presented in this article, the consideration of adopting restrictions in the circulation of vehicles may be supported by findings in this study.

The epidemiological context and results of the second detected change-point was vastly different from the first period. The analysis showed an increase of 387% in the number of new daily cases with respect to the projection by the BTS analysis. Before 20 June 2020, the country started the gradual reopening of commercial activities, as the number of new cases was stabilizing, and health officials had started to increase hospital capacity. These measures started on 1 May 2020 [14], divided into periods of 14 days each and involved the gradual opening of businesses such as gymnasiums, movie theaters, beauty salons, hotels, as well as the opening of beaches with restrictive schedules, and national parks [14].

Overall, during this second period of the analysis, although mobility dynamics were positively correlated with easing public health measures, the results of the Pillai test showed a weaker conditional effect compared to the first period of analysis. This can be expected due to the complex interaction of the other social, economic, and behavioral factors in this period. For example, besides the gradual reopening of commerce, the country was also starting to witness an increase in the number of new cases detected in areas located near the northern border of Costa Rica with Nicaragua. The cases were mostly linked with clusters being reported in locations with agricultural activities and processing factories [14], which typically receive a significant number of migrant workers who cross illegally, despite the border being closed. These phenomena may have limited the capacity of the models to accurately associate the increase in daily new cases with the public health measures implemented during that period.

Our findings of 7–8 days lag effect on infections after the onset of mobility reduction contrast with those of Cot et al. [11], who reported 2–5 weeks and are closer to the 9–12 days described by Badr et al. [10]. Our numbers are consistent with the incubation time of SARS-CoV-2, which may reflect a more direct effect on the mobility measures captured in our study. Our reduction effect on infection rates of 54% is about the average of what Cot et al. [11] found for Europe and the United States and are comparable to the 35% and 63% reported by Badr et al. in [10].

We remark that one of the limitations of the study is that mobility data is aggregated at a national level. Therefore, it was not possible to analyze differences in mobility by region. Moreover, the statistical analysis relied on a series of assumptions. For example, the use of a single midpoint to represent each set of measures can change their real effect over the mobility series, or the effect not accounted for by the mobility data on the detected change-points can have more temporal structure than the one assumed in Equation (1). The study also has limitations on capturing the effect of behavioral changes associated with sanitary measures in different periods of the pandemic. The imposition of restrictions in the early stages of the disease might have had a larger impact due to the fear in the general population of a new deadly virus. The widespread use of personal protective measures such as face masks also changed during the pandemic.

Despite these limitations, results from our study provide an insight into the critical role of sanitary measures in controlling the spread of the virus in the initial phases, when countries and health systems are preparing to receive a surge in severe cases. It is important to communicate these results and their conclusions to public health experts and decision makers currently battling to control the pandemic and to those in charge of preparing for future (similar) events.

Further research should explore the effect of human mobility and the role that sanitary measures play in the context of vaccination roll-outs and the emergence of new variants of SARS-CoV-2. Beyond the elevated death toll of COVID-19, the cost of having had an international community poorly prepared to confront the challenges of the pandemic prior to 2020 will bring serious and lasting consequences to the whole world, some of which we are only starting to understand. In an effort to learn our lessons, it is important to study which measures have been effective to contain, control, or mitigate the spread of the disease. This study can contribute to being better prepared for future pandemics but can also inform on tools to transition out of the current situation.

## Figures and Tables

**Table 1:** Significant cross-correlation lags under a Pearson test. The remaining categories show no-significant lags.

Categories	Notation in (1)	Lag (Days)
Retail and Recreation	$M_{t}^{\left(1\right)}$	7
Parks	$M_{t}^{\left(2\right)}$	7
Transit stations	$M_{t}^{\left(3\right)}$	7
Residential	$M_{t}^{\left(4\right)}$	8

**Table 2:** Sanitary and mobility measures from March through June, 2020. The *p*-value of the Pillai test is shown in the fourth column.

Set	Date	Measures	Pillai Test
1	3/10–3/22	Cancellation of massive events and work from home instructions; closure of bars and casinos; Government declares National Emergency; Closure of all schools and borders; Closure of movies, theaters, and gymnasiums, malls at 50%.	<2.2 × 10^−16^
2	3/23–4/2	Closure of beaches, churches, and national parks; Start of vehicle restrictions from 10 p.m. to 5 a.m.; Vehicle restriction on weekends from 8 p.m. to 5 a.m.; Closure of non-essential commercial activities starting at 8 p.m. and during the weekend.	8.94 × 10^−14^
3	4/3–4/7	Daytime vehicle restriction from 5 a.m. to 5 p.m., with plate distribution and nighttime vehicle restriction from 5 p.m. to 5 a.m.; Restriction of long-distance public transportation; Closure of non-essential commercial activities.	2.38 × 10^−3^
4	4/8–4/12	The circulation of vehicles and public transportation was suspended.	2.71 × 10^−11^
5	4/13–4/30	Day-time vehicle restriction from 5 a.m. to 7 p.m. with plate distribution; Total nighttime vehicle restriction from 7 p.m. to 5 a.m.; On weekends, total vehicle restriction (with exceptions to access to essential services); Commerce may function from 5 a.m. to 7 p.m., weekends delivery only.	9.65 × 10^−10^
6	5/1–5/15	The first phase of gradual reopening of commercial activities; Vehicle restriction from 5 a.m. to 7 p.m. with plate distribution; Total night-time restriction from 7 p.m. to 5 a.m.	2.01 × 10^−7^
7	5/16–5/31	The second phase of the gradual reopening of commercial activities; Vehicle restriction from 5 a.m. to 10 p.m. with plate distribution; Total nighttime restriction from 10 p.m. to 5 a.m.	2.03 × 10^−5^
8	6/1–6/19	The third phase of the gradual reopening of commercial activities; Vehicle restriction from 5 a.m. to 10 p.m. with plate distribution; Total nighttime restriction from 10 p.m. to 5 a.m.; Differentiated vehicle restriction in municipalities located near the northern border area of Costa Rica.	8.84 × 10^−4^
9	6/20–6/21	Total vehicle restriction with circulation only to essential services; Closure of non-essential commercial activities.	8.84 × 10^−4^

## Data Availability

The data used in this paper is available at COVID-19 Community Mobility Reports. Available online: https://www.google.com/covid19/mobility/ (accessed on 19 July 2021). Data of Covid-19 cases is available at the Ministry of Health web page http://geovision.uned.ac.cr/oges/index.html (accessed on 19 July 2021).
